# Comparison of Diagnostic Methods for Respiratory Disease in Calves Used on Farms With Thoracic Radiography

**DOI:** 10.1155/vmi/5539202

**Published:** 2025-04-23

**Authors:** João Paulo Andrade, Gabriela Anteveli, Bárbara de Andrade Alves, Layanne D. Ferreira, Filipe L. M. Mendonça, Rafael J. Silva, Jerusa Catarina Camillo, Markus V. V. Araújo, Luana C. A. Ferreira, Anelise Carvalho Nepomuceno, Rafael Resende Faleiros, Tiago Facury Moreira, Elias Jorge Facury Filho, Rodrigo Melo Meneses, Antônio Último de Carvalho

**Affiliations:** Department of Veterinary Clinics and Surgery, Federal University of Minas Gerais, Belo Horizonte, Minas Gerais, Brazil

**Keywords:** California respiratory score, pulmonary auscultation, pulmonary ultrasound, thoracic radiography, Wisconsin respiratory score

## Abstract

The most commonly used techniques in the field are the pulmonary auscultation, Wisconsin score (WI), California score (CA), and pulmonary ultrasonography. However, with the exception of the latter, no studies have compared thoracic radiography with other possible techniques in calves. Therefore, the objective of the study was to compare and verify the agreement between clinical score techniques, pulmonary auscultation, and ultrasonographic and radiographic evaluations, considering the latter as reference test. Thirty-three calves were evaluated from 17 to 60 days of age using pulmonary auscultation, Wisconsin score (WI), California score (CA), thoracic radiography, and pulmonary ultrasonography at five preestablished moments and at any time when presenting clinical respiratory disease. Of the 160 evaluations, 21% were positive for thoracic radiography, 21% for ultrasonography, 10% for pulmonary auscultation, 16% for CA score, and 14% for WI score. In the concordance analysis, there was a moderate correlation between thoracic radiography and ultrasonography (*k* = 0.6035) and between pulmonary auscultation and WI score (*k* = 0.5833) and CA score (*k* = 0.5277), and substantial between the WI and CA score methods (*k* = 0.7258). All techniques used in the study were useful for the diagnosis of pneumonia; however, due to high accuracy and practicality, ultrasonography proved to be an interesting method to be implemented on farms.

## 1. Introduction

Pneumonia is an inflammatory response of the body that can be caused by some microorganisms. Bovine respiratory disease (BRD) is a group of infectious diseases that affect the respiratory system and is responsible for a large proportion of deaths in nursing and weaned calves [1]. In addition to causing mortality, heifers with bronchopneumonia during the nursing and weaning phases may have lower weight gain, increased age at first calving, and lower milk production [2–4].

One of the major challenges for adequate control and treatment of BRD is the difficulty in diagnosis, especially in subclinical and chronic cases [5–7]. Therefore, in recent decades, research has sought more efficient and early diagnostic methods that can be applied to the daily routines of farms. These methods include pulmonary auscultation (AUS) and respiratory scores, with the most commonly used being the Wisconsin score (WI) [1], the California score (CA) [8], and pulmonary ultrasound (TUS) [9].

However, each of these methods has limitations. When used individually, scoring systems may have low sensitivity (46%–64%) and moderate to high specificity (74.1%–91.2%) [8, 10]. Pulmonary auscultation, despite being simple and quick, is highly dependent on the examiner's experience and has high subjectivity, and not every animal with lung damage shows changes in respiratory sounds [6, 11, 12]. Ultrasound of the lungs has been routinely used in the diagnosis of DRB, with good sensitivity and specificity (94% and 100%, respectively) [13], and demonstrates the ability to detect early lesions and subclinical conditions [9, 14].

Despite the methods described above being widely used in calves under farm conditions and in human and small animal medicine, thoracic radiography is considered one of the most sensitive methods and is widely used in diagnosing lung diseases [15, 16]. A few studies that compared the detection ability of ultrasound and radiography for lung diseases in calves found a good correlation [9]. Therefore, this study aimed to compare thoracic radiography with clinical scores, pulmonary auscultation, and ultrasonographic evaluations.

## 2. Materials and Methods

### 2.1. Animals

The activities developed in this study were submitted and approved by the Ethics Committee on Animal Use (ECAU) of UFMG under the protocol of the number 245/2016.

In this study, 33 calves from a commercial farm were transported to the study location on the day after birth. From 3 to 10 days of age, the animals remained in pens with sawdust bedding in groups of two to four animals, where they received feeding and umbilical cord care twice a day. From the 10th day of life, they were transferred to a tie stall with rubber matting, individual feed troughs, and water. The facility has fans that are turned on during the day.

The animals were fed with 6 L of reconstituted milk (130 g of powdered milk [Itambé®] for every 870 mL of water) up to 45 days of age, divided into two meals of 3 L. From 46 to 60 days, they received 3 L with a single feeding at 8 a.m.; from 10 days of age, they were provided hay and feed (18% crude protein).

### 2.2. Physical Examination and Diagnosis of BRD

The animals were monitored from 17 to 59 days of age with the physical examination of the animals that was performed daily, evaluating heart rate, respiratory rate, rectal temperature, lung auscultation, and respiratory scores using the Wisconsin (WI) [1] and California (CA) scoring systems [8].

Thoracic radiography and pulmonary ultrasonography were performed on all calves at five predetermined time points (17-18, 24-25, 31–34, 45-46, and 59-60 days of age) and at any time during the experiment when the animal presented any clinical signs associated with BRD on physical examination (fever, lung crackles, changes in respiratory pattern, reduced food intake, and prostration).

The pulmonary auscultation was performed using a stethoscope (Littmann Classic III®) positioned over the lung area, following the method described by Dirksen et al. [17], in which the lung area was examined in a line shape in the ventro-dorsal direction, and at least two respiratory movements were auscultated in each focus.

The pulmonary ultrasonography was performed in a caudo-cranial direction, from the 10th to the third intercostal space, and in each of these spaces, it was evaluated from the dorsal to the ventral limit, according to Rabeling et al. [18]. The equipment used was a Mindray DP-2200VT® with a convex transducer at a frequency of 5 MHz, image depth of 8.4–12 cm, and evaluation in the B-mode.

Pulmonary consolidations were measured according to the extent of consolidation using the measurement (measure) feature of the ultrasound equipment on the frozen image (freeze). The ultrasonographic findings were classified according to Buczinski et al. [6]. Briefly, Grade 0 is lung without alteration or with a few comet tails; Grade 1 is countless comet tails; Grade 2 is consolidation (hyperechoic area) less than 1 cm; Grade 3 is consolidation between 1 and 2 cm; Grade 4 is consolidation between 2 and 3 cm; and Grade 5 is consolidation greater than 3 cm. The animals were considered to have lung disease when they presented pulmonary consolidation greater than 1 cm in depth.

The thoracic radiograph was performed using the Veterinary Portable X-Ray machine (Orange® 1040HF Vet 40–100 kv/0.32–100 m) adjusted to 2.5 mAs and 100 kV, with the center of the image positioned between the third and fourth intercostal spaces. The radiographic cassette was digitized using the Regius Model 110 device. During analysis, each radiograph was classified according to the observed characteristics in normal, interstitial, bronchial, and alveolar radiographic patterns, as described by Thrall and Widmer [19]. Animals that presented with interstitial, bronchial, or alveolar patterns were considered to have lung disease.

All animals diagnosed with clinical pneumonia, presence of lung crackles, increased respiratory rate, fever, behavioral changes with reduced food intake and lethargy, or any other illness during the experiment were treated with an appropriate therapeutic protocol according to Divers and Peek [20]. The medications used were penicillin (30,000 IU/kg/IM) for 5 days or enrofloxacin (7.5 mg/kg/IM) in a single dose. In both cases, flunixin meglumine (1.1 mg/kg/IM) was administered for 3 days.

Calves that showed deterioration in their clinical condition and did not respond to treatment were euthanized.

### 2.3. Statistical Analyses

The results of the AUS evaluations, WI score, CA score, pulmonary ultrasonography, and thoracic radiography were analyzed descriptively to assess the correspondence between positive cases, frequency of animals, and number of positive evaluations by technique. To compare the efficiency of the techniques, thoracic radiography was considered the “standard” test, and the sensitivity, specificity, positive predictive value, and negative predictive value were calculated for AUS, WI score, CA score, and pulmonary ultrasonography, following Dohoo et al. [21].

The Kappa method described by Cohen [22] was used to determine the correlation between the techniques of AUS, WI score, CA score, pulmonary ultrasonography, and thoracic radiography, and the classification criteria of *k* values proposed by Landis and Koch [23] were used.

## 3. Results

### 3.1. Agreement Analysis of Techniques

Of the 33 calves that started the experiment, four died and five evaluations were lost; thus, a total of 160 evaluations were performed per technique. Of these, 21% (33/160) were positive on thoracic radiography (TRX), 21% (34/160) on pulmonary ultrasonography (TUS), 16% (25/160) on CA score, 14% (22/160) on WI score, and 10% (16/160) on pulmonary auscultation (AUS). When analyzed per animal, 52% (17/33) presented at least one positive evaluation for pulmonary disease in the pulmonary ultrasonography exam, 55% (18/33) in the thoracic X-ray, 40% (13/33) in pulmonary auscultation, 42% (14/33) in the WI score, and 52% (17/33) in the CA score. When evaluating the results together from the five techniques used, 67% (22/33) of the animals were positive in at least one evaluation using any of the five techniques.

In the agreement analysis (Table 1), moderate agreement was found between the thoracic X-ray and pulmonary ultrasonography techniques (0.6035) and substantial agreement was found between the WI and CA methods (0.7258). Pulmonary auscultation had a moderate correlation with clinical scores (0.5833; 0.5277), unlike imaging techniques, which had weak to slight agreement with clinical methods, according to the classification of Kappa values proposed by Landis and Koch [23].

### 3.2. The Efficiency Level of the Techniques Considering Thoracic Radiography as the Reference for the Pulmonary Disease Diagnosis

Considering thoracic radiography as the standard for pulmonary disease diagnosis, the sensitivity of pulmonary ultrasound was 70%, pulmonary auscultation was 33%, the WI score was 36%, and the CA score was 27%. The specificities of these techniques were 91%, 96%, 92%, and 87%, respectively (Table 2).

When comparing positive cases in radiographic patterns (Table 3), alveolar pattern (AP-17 cases) and interstitial pattern/bronchial pattern (IP/BP-11 and 4 cases, respectively), with the other diagnostic techniques employed, TUS was positive in 100% (17/17) of cases when AP was identified and 52.9% (9/17) of cases with IP/BP pattern. The other techniques had correspondence values ranging from 41.1% to 58.8% for AP and 12.5% for IP/BP for all methods. Additionally, animals with AP had higher respiratory frequency and rectal temperature values.

### 3.3. Necropsy Findings

During the study, four animals died, one from sepsis and three due to pulmonary complications, all of which tested positive in all techniques in the last antemortem evaluation. The macroscopic analysis revealed that the lesions were similar to the consolidation sites assessed by pulmonary ultrasound and exhibited an alveolar pattern on chest radiography. However, lesions extending into the cranial portion of the left and right cranial lobes were not diagnosed using pulmonary ultrasound.

## 4. Discussion

Radiographic examination is still widely used as a means of diagnosing respiratory diseases in humans, despite having some limitations [15, 16]. In cattle, radiography for diagnosing lung diseases is seldom used in practice or research [24, 25]. Several studies have adopted and advocated the use of ultrasound as an efficient method for diagnosing respiratory diseases in calves [26]. These studies have also compared ultrasound with auscultation techniques and scoring methods [6], but few studies have compared TUS and TRX techniques [9], while none have compared TRX with TUS, AUS, WI, and CA.

Ultrasound and radiographic techniques were superior to auscultation and clinical scores in detecting the lung disease in calves. This may be because imaging tests can detect pulmonary changes in both clinical and subclinical cases [14]. In addition, the parameters used in the scores are less specific and may be altered in upper respiratory tract infections, causing the animal to test positive without necessarily having any lung disease [14, 27].

When comparing different diagnostic methods with TRX, we observed low sensitivity (< 36%) for AUS, WI, and CA, and substantial sensitivity for TUS (70%), while the specificity of all methods was high (> 87%). However, when we separated TRX according to its pattern (alveolar, bronchiolar, or interstitial), we noticed that the sensitivity and specificity of the methods could be considerably higher if only the radiographic exams with alveolar pattern were considered. To give an idea, TUS identified 100% of the TRX exams with alveolar pattern, while AUS and WI and CA identified 52.9%, 58.8%, and 41.1% of the positive results in TRX in alveolar patterns. Similarly, Berman et al. [9] found almost identical results when using TUS and TRX, as all calves presented alveolar patterns in TRX.

Our results also demonstrated that the alveolar pattern is more correlated with clinical diseases, which is consistent with reports of this type of pattern in severe viral or bacterial infections [25, 28].

The low sensitivity of interstitial and bronchiolar pattern detection in AUS and clinical scoring techniques can probably be explained by the fact that they mostly represent mild cases without clinical changes and without intense inflammation of the bronchial network, and therefore without changes in lung sounds. In addition, in cases of TRX with an interstitial pattern, the alveolar lumen remains filled with air, and the reverberation artifact formed by ultrasound is similar to that in a healthy lung [29].

Although comet tails are related to thickening of the lung interstitial, in the present study, only consolidations greater than 1 cm were considered indicative of lung disease according to Buczinski et al. [6]. If comet tails were considered as a lung disease, it would increase the sensitivity of TUS by increasing the number of positive cases but would reduce its specificity. In cattle and human medicine, B-lines (comet tails) artifacts have been reported to be associated with interstitial pneumonia [30–32], but they are not very specific and can occur in healthy lungs, pulmonary edema, irregularities in the pleura, and areas with atelectasis [6, 11, 18, 29, 30].

When comparing the clinical methods with TUS and TRX, the correlation was mild to weak. This is because calves with lung consolidations may not present clinical signs and may not be identified by WI, CA, or AUS [6, 13]. The correlations between AUS and the WI and CA systems were moderate, as observed by Gouda [26]. This correlation can be understood by the use of hyperthermia and increased respiratory rate as parameters in the score systems.

As expected, the WI and CA scores showed high agreement with each other because many of the parameters used were the same [33]. Despite the high agreement, CA showed slightly higher sensitivity and specificity. In CA, the intensity of the signs is not considered, while in WI it is. Thus, changes in two of the evaluated parameters can result in a positive score in WI, and most of the time, CA requires three altered parameters. In these cases, WI may increase the number of false positive cases for lung disease.

The necropsy findings reinforced the positive results of this study, showing that three animals that died from pulmonary complications tested positive in clinical tests and imaging methods. However, the lesions were more extensive than those assessed in the ultrasound image because the probe used did not allow visualization of the cranial portions of the cranial lobes, and they died days after the last evaluation, contributing to the extension of the lesion.

When the objective is the comparison between different diagnostic methods, a reference test with high accuracy should be employed. However, in the case of BRD, a test with 100% accuracy does not yet exist [12]. Consequently, a limitation of the study was the failure to account for the potential shortcomings of thoracic radiography, the reference technique utilized in the current research. Nonetheless, it is important to highlight that the selection of thoracic radiography was based on the high sensitivity and specificity reported in previous studies [24, 25], and due to its widespread use as a confirmatory test in both human medicine and small animal medicine [15, 16]. For future studies, where suitable for the research material, it is recommended to employ Bayesian analysis as a means to mitigate the bias of the techniques used.

Finally, our study reinforces that pulmonary ultrasonography presents itself as a promising alternative, as it has good sensitivity and high specificity compared to thoracic radiography in cases of alveolar patterns. Since evaluations with interstitial/bronchial patterns had low clinical importance, we believe that pulmonary ultrasonography is sufficiently accurate to detect lung diseases in calves. Additionally, pulmonary auscultation and the Wisconsin and California scores have moderate sensitivity (< 36%) and high specificity when compared to thoracic radiography. Therefore, the association of one of the imaging techniques with clinical techniques should be implemented to achieve a more effective diagnosis of lung diseases.

## 5. Conclusion

Pulmonary ultrasonography has good sensitivity and high specificity compared with thoracic radiography in cases of alveolar patterns. As evaluations with interstitial/bronchial pattern had low clinical importance, we believe that pulmonary ultrasonography is accurate enough to detect lung disease in calves. Pulmonary auscultation and Wisconsin and California scores have moderate sensitivity (< 36%) and high specificity when compared to thoracic radiography. The association of one of the imaging techniques with clinical techniques should be conducted to have a more effective diagnosis of lung disease.

## Figures and Tables

**Table 1:** Concordance relationships between thoracic radiography (TRX), pulmonary ultrasound (TUS), pulmonary auscultation (AUS), and clinical scores of Wisconsin (WI) and California (CA).

	TRX	TUS	AUS	WI	CA
TRX	1.0000	—	—	—	—
TUS	0.6035	1.0000	—	—	—
AUS	0.3632	0.3297	1.0000	—	—
WI	0.3249	0.3569	0.5833	1.0000	—
CA	0.1830	0.2764	0.5277	0.7258	1.0000

**Table 2:** Sensitivity (Se), specificity (Sp), positive predictive value (PPV), and negative predictive value (NPV) of TUS, AUS, WI, and CA compared with TRX evaluations.

		TRX		Se	Sp	PPV	NPV
		Positive	Negative				
TUS	Positive	23	11	70%	91%	68%	92%
	Negative	10	116				
	Total	33	127				

AUS	Positive	11	5	33%	96%	69%	84%
	Negative	22	122				
	Total	33	127				

WI	Positive	12	10	36%	92%	55%	85%
	Negative	21	117				
	Total	33	127				

CA	Positive	9	16	27%	87%	36%	82%
	Negative	24	111				
	Total	33	127				

**Table 3:** Positive evaluations of TRX classified into interstitial/bronchial patterns (IP/BP) and alveolar patterns (AP) and their correspondence in TUS, AUS, WI, and CA.

Patterns	Positives	TUS	AUS	WI	CA
IP/BP	16	37.5% (6/16)	12.5% (2/16)	12.5% (2/16)	12.5% (2/16)
AP	17	100% (17/17)	52.9% (9/17)	58.8% (10/17)	41.1% (7/17)
Total	33	69.7% (23/33)	30.3% (11/33)	36.3% (12/33)	27.2% (9/33)

## Data Availability

The datasets used and analyzed during the current study are available from the corresponding author upon request.
